# Identifying potential prognosis markers in hepatocellular carcinoma *via* integrated bioinformatics analysis and biological experiments

**DOI:** 10.3389/fgene.2022.942454

**Published:** 2022-07-19

**Authors:** Xueting Hu, Jian Zhou, Yan Zhang, Yindi Zeng, Guitao Jie, Sheng Wang, Aixiang Yang, Menghui Zhang

**Affiliations:** ^1^ Department of Intensive Care Unit, The Affiliated Suzhou Hospital of Nanjing Medical University, Suzhou Municipal Hospital, Gusu School, Nanjing Medical University, Suzhou, Jiangsu, China; ^2^ Department of Hematology, The Affiliated Hospital of Xuzhou Medical University, Xuzhou, Jiangsu, China; ^3^ Department of Hematology, Linyi Central Hospital, Yishui, Shandong, China

**Keywords:** hepatocellular carcinoma, bioinformatics, RRM2, osalmid, pathway

## Abstract

**Background:** Hepatocellular carcinoma is one kind of clinical common malignant tumor with a poor prognosis, and its pathogenesis remains to be clarified urgently. This study was performed to elucidate key genes involving HCC by bioinformatics analysis and experimental evaluation.

**Methods:** We identified common differentially expressed genes (DEGs) based on gene expression profile data of GSE60502 and GSE84402 from the Gene Expression Omnibus (GEO) database. Gene Ontology enrichment analysis (GO), Kyoto Encyclopedia of Genes and Genomes (KEGG) pathway analysis, REACTOME pathway enrichment analysis, and Gene Set Enrichment Analysis (GSEA) were used to analyze functions of these genes. The protein-protein interaction (PPI) network was constructed using Cytoscape software based on the STRING database, and Molecular Complex Detection (MCODE) was used to pick out two significant modules. Hub genes, screened by the CytoHubba plug-in, were validated by Gene Expression Profiling Interactive Analysis (GEPIA) and the Human Protein Atlas (HPA) database. Then, the correlation between hub genes expression and immune cell infiltration was evaluated by Tumor IMmune Estimation Resource (TIMER) database, and the prognostic values were analyzed by Kaplan-Meier plotter. Finally, biological experiments were performed to illustrate the functions of RRM2.

**Results:** Through integrated bioinformatics analysis, we found that the upregulated DEGs were related to cell cycle and cell division, while the downregulated DEGs were associated with various metabolic processes and complement cascade. RRM2, MAD2L1, MELK, NCAPG, and ASPM, selected as hub genes, were all correlated with poor overall prognosis in HCC. The novel RRM2 inhibitor osalmid had anti-tumor activity, including inhibiting proliferation and migration, promoting cell apoptosis, blocking cell cycle, and inducing DNA damage of HCC cells.

**Conclusion:** The critical pathways and hub genes in HCC progression were screened out, and targeting RRM2 contributed to developing new therapeutic strategies for HCC.

## Introduction

Hepatocellular carcinoma (HCC) is malignant cancer with high morbidity and mortality, which is the fourth leading cause of cancer-related deaths globally (Ichikawa et al., 2014; Wang and Zhang, 2020). In recent years, there have been significant improvements in non-drug therapies and drug therapies for HCC patients, including hepatic resection, liver transplantation, chemoembolization (TACE), and targeted therapy, which exhibit notably improved survival and prognosis in patients with HCC (El-Serag and Rudolph, 2007; Chen et al., 2020). There is an increasing body of evidence that abnormal expression of genes and mutations of tumor-suppressor genes are associated with mechanisms of HCC tumorigenesis and progression, including cyclin D1 (CCND1), pre-mRNA processing factor 3 (PRPF3), c-Myc or Ras and p53 (Choi et al., 2001; Wang et al., 2012; Li et al., 2017a; Liu et al., 2020). However, due to treatment resistance and post-surgical recurrence, the therapeutic outcomes have not been effective as expected, and the molecular mechanisms of liver carcinogenesis remain unclear (Wong et al., 2018; Chen et al., 2019; Song et al., 2020). Thus, it is urgent to identify novel biomarkers and therapeutic targets for early diagnosis and individualized treatment.

Currently, genomic microarrays and high-throughput sequencing technology have become reliable methods to explore molecular markers, coupled with bioinformatics analysis (Liu et al., 2019; Gao et al., 2020; Wang et al., 2020). In bioinformatics, the public databases such as Gene Expression Omnibus (GEO), The Cancer Genome Atlas (TCGA), Gene Expression Profiling Interactive Analysis (GEPIA) and Human Protein Atlas (HPA) are commonly used databases, and Kaplan-Meier plotter is a powerful tool to analyze the prognostic value of genes. Through these technologies, many researchers have found the hub genes related to progression, diagnosis, and prognosis of HCC (Lin et al., 2019; Shen et al., 2019; Song et al., 2020). Nonetheless, these studies are only based on single analyses without experimental validation. To overcome this disadvantage, integrated bioinformatics methods should be combined with experiments.

In the present study, the microarray datasets GSE60502 and GSE84402 were obtained from the GEO database, which included 32 hepatocellular carcinoma tissues and 32 adjacent non-tumorous liver tissues. The differentially expressed genes (DEGs) were identified using the online tool GEO2R and analyzed with bioinformatics methods such as the Database for Annotation, Visualization, and Integrated Discovery (DAVID), REACTOME, and GSEA. Then, we constructed the protein-protein interaction (PPI) network for module analysis and hub genes identification in HCC. Expression validation, immune infiltration analysis, and survival analysis of the hub genes were performed by GEPIA, HPA, TIMER, and Kaplan Meier plotter. Finally, we determined the effects of RRM2 inhibitor osalmid on proliferation, cell apoptosis, cell cycle, migration, and DNA damage *in vitro*.

## Materials and methods

### Data collection and data processing

We obtained Gene expression profile data of HCC patients from the GEO database (https://www.ncbi.nlm.nih.gov/geo/). The GSE60502 dataset contained 18 HCC tissues and 18 adjacent non-tumorous liver tissues, while the GSE84402 dataset included 14 HCC tissues and 14 adjacent non-tumorous liver tissues. The microarray data of GSE60502 are based on GPL96 platforms (HG‐U133A Affymetrix Human Genome U133A Array) and the GSE84402 data are based on GPL570 platforms [HG-U133_Plus_2] Affymetrix Human Genome U133 Plus 2.0 Array.

GEO2R online tools were used to identify DEGs between HCC tissues and normal hepatic tissues. Genes that met the specific cut-off criteria of P-value < 0.05 and |logFC|>2 were considered as DEGs. The intersecting genes of the two GEO datasets were examined using the Venn diagram web tool.

### Gene ontology annotation and pathway enrichment analysis

To reveal the functions of the above genes, we conducted GO annotation and KEGG pathway enrichment analysis via the DAVID database (Huang et al., 2009). The GO terms contained biological process (BP), cellular component (CC), and molecular function (MF). We also used another online database, REACTOME, to analyze pathways (Jassal et al., 2020). P-value < 0.05 was considered statistically significant.

### Gene set enrichment analysis

GSEA was performed to predict biological function and related signaling pathways of genes in these two datasets (Subramanian et al., 2005). Annotated gene sets c2.cp.kegg.v7.4. symbols.gmt were chosen as the reference gene sets. *p*-value < 0.05 and FDR <0.25 were set as the cut-off criteria.

### Construction of protein-protein interaction network

The STRING database was used to build a protein-protein interaction network of DEGs, and all PPI pairs with a combined score of >0.4 were extracted (Szklarczyk et al., 2019). We used Cytoscape to visualize the PPI network, and Module analysis was conducted utilizing MCODE, a plug-in in Cytoscape (Shannon et al., 2003). The rank methods of Density of Maximum Neighborhood Component (DMNC), Maximum Neighborhood Component (MNC), Closeness (Node connect closeness), EPC (Edge percolated component), and Degree (Node connect degree) in plug-in CytoHubba were utilized to determine the hub genes (Chin et al., 2014; Yang et al., 2019; Gao et al., 2020).

### Validation of hub genes

The mRNA expression levels of the hub genes in human normal and HCC tissues were determined using the GEPIA database, including data of 369 tumors and 160 normal samples from the TCGA and GTEx projects (Tang et al., 2017). For further validation of protein expression, we utilized the immunohistochemistry (IHC) database HPA to confirm it (Asplund et al., 2012).

### Immune infiltration analysis and survival analysis

To explore whether these genes were related to immune infiltration, we used the TIMER database to evaluate the correlation between prognostic gene expression and immune cell infiltration (Li et al., 2017b). Kaplan-Meier plotter was utilized to perform survival analysis of the previously identified hub genes, an online database containing clinical and gene expression data (Lánczky and Győrffy, 2021). The patient samples were split into two groups based on the median expression of the gene, assessing the prognostic value of a specific gene.

### Cell culture and agents

The human HCC cell lines HepG2 and Hep3B were obtained from the American Type Culture Collection (ATCC). All cells were cultured in a DMEM medium (KeyGEN, China) contained with 10% FBS at 37°C, 5% CO_2_, and they were used for subsequent experiments in the logarithmic growth phase. Osalmid purchased from MCE was a ribonucleotide reductase small subunit M2 (RRM2) targeting compound (Liu et al., 2016).

### Cell counting Kit-8 assay

We used a Cell counting Kit-8 (KeyGEN, China) to access cell proliferation and viability. The HCC cells were plated in 96-well plates at a density of 2.5 × 10^3^ per well with 100 μl of culture medium, treated with osalmid at different concentrations for 48 h.

### Wound-healing assay

The Hep3B cells were counted and cultured in 6-well culture plates and put in the incubator overnight. After 24 h, the density of cells could be close to 100%, we scratched the monolayer with a micropipette tip and photographed it. Then we treated cells with different doses of osalmid and put the 6-well plates back to the incubator for 48 h. Then the images of the scratching areas were compared.

### Transwell migration assay

Cell migration ability was assessed using Transwell assay. We added 4 × 10^4^ Hep3B cells and 200 μl DMEM medium supplemented with 10% FBS into the upper chamber with a microporous (8.0 µm pores) transwell insert (Corning Incorporated). The bottom chamber was filled with DMEM medium containing 20% FBS. While incubation treated with different concentrations of osalmid for 48 h, the cells in the upper chamber migrated through the Matrigel Matrix (Corning, 356234)-coated porous membrane to the lower chamber to some extent. Then these cells were stained with 0.1% crystal violet for 6 min and randomly selected five fields were photographed at a magnification of ×10.

### Flow cytometry

Cell apoptosis and cell cycle were analyzed by flow cytometry. HCC cells were seeded into 6-well at approximately 30% density treated with different concentrations of osalmid. Cell apoptosis was determined by an Annexin V-APC/7-AAD Detection Kit (KeyGEN, China), by the manufacturer’s protocol. For the cell cycle, the HCC cells were fixed with 70% ethanol at 4°C overnight. On the next day, the cell cycle was examined using Cell Cycle Staining Kit (KeyGEN, China) according to the manufacturer’s instructions.

### Western blot

The western blot analysis was conducted as described previously (Yao et al., 2014). The following antibodies were used: β-actin, CDK2, PARP1 (Proteintech), cyclinA2 (Cell Signaling Technology), and γH2AX (ABclonal).

### Statistical analysis

All results are presented as means ± standard deviation (SD). The student’s t-test was used to determine significant differences between the two groups. All P values less than 0.05 were considered to be statistically significant.

## Results

### Identification of differentially expressed genes in hepatocellular carcinoma

**FIGURE 1:**
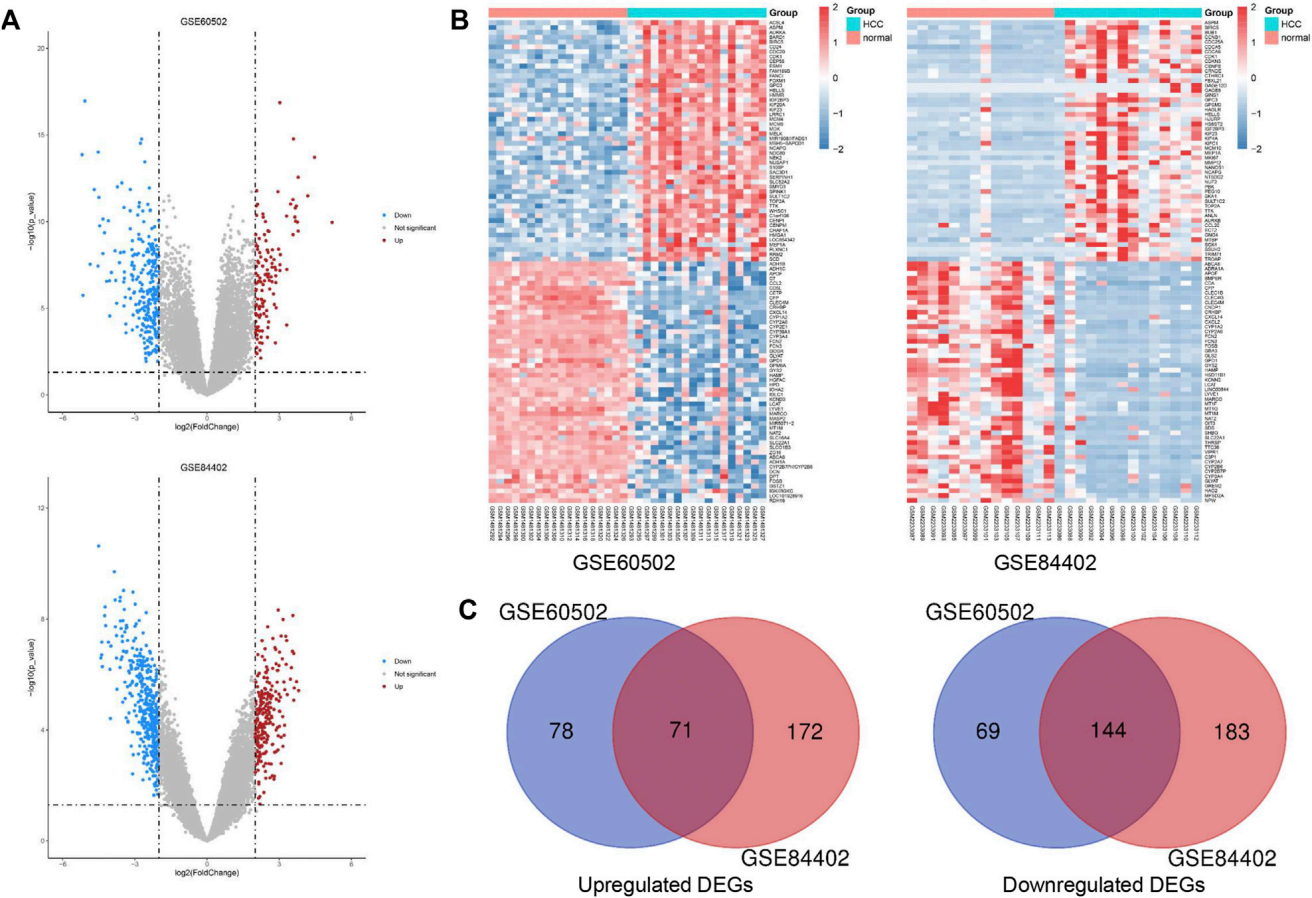
Identification of common DEGs in two cohort profile datasets (GSE60502 and GSE84402). **(A)** Volcano plots of the two datasets. **(B)** The heatmap of the top 100 DEGs. Z-score was from −2 to 2. **(C)** Venn diagram of downregulated and upregulated DEGs based on the two GEO datasets.

We obtained 149 upregulated and 213 downregulated genes following the GSE60502 dataset analysis, and 243 upregulated and 327 downregulated genes from the GSE84402 dataset (Figure 1A). The heatmap showed the top 100 DEGs in these two datasets, including 50 upregulated and 50 downregulated DEGs (Figure 1B). A total of 215 genes were identified as common DEGs, including 71 upregulated and 144 downregulated genes (Figure 1C and Supplementary Figure S1).

### Gene ontology enrichment analysis of differentially expressed genes

**FIGURE 2:**
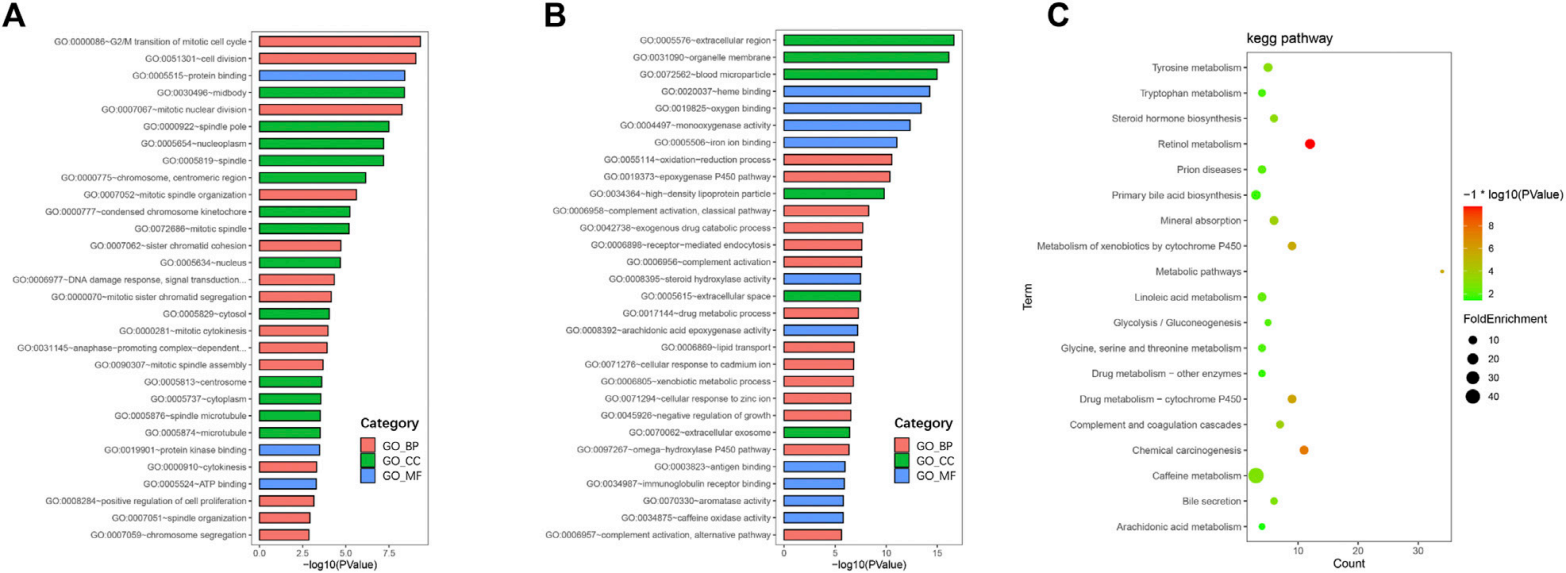
GO and KEGG pathway enrichment analyses. GO enrichment analysis of upregulated **(A)** and downregulated **(B)** DEGs consisting of biological process (BP), molecular function (MF), and cellular component (CC). **(C)** Bubble plot of KEGG pathway enrichment analysis of downregulated DEGs.

To further understand the functions and mechanisms of these identified DEGs, we used the DAVID online tool to conduct GO annotation enrichment analysis (Figures 2A,B). The BP category of GO analysis results indicated that upregulated genes were enriched in the G2/M transition of mitotic, cell division, mitotic nuclear division, and mitotic spindle organization, and downregulated genes were enriched in oxidation-reduction process, epoxygenase P450 pathway, complement activation, and exogenous drug catabolic process. Moreover, upregulated genes in CC were mainly involved in the midbody, spindle pole, and nucleoplasm, and the downregulated genes were mainly in the extracellular region, organelle membrane, and blood microparticle. In addition, for MF, enrichment of upregulated genes was primarily in protein binding, protein kinase binding, and ATP binding, and that of downregulated genes was primarily in heme binding, oxygen binding, and monooxygenase activity.

### Signaling pathway enrichment analysis

KEGG pathway enrichment analysis showed that upregulated DEGs were enriched in the cell cycle, p53 signaling pathway, oocyte meiosis, and progesterone-mediated oocyte maturation (Table 1). The downregulated DEGs were mainly enriched in caffeine metabolism, retinol metabolism, metabolic pathways, drug metabolism-cytochrome P450, and complement and coagulation cascades (Figure 2C). Also, REACTOME pathway enrichment analysis was performed, whose result was consistent with the KEGG enrichment analysis result (Table 2). The functions of upregulated DEGs were closely related to cell cycle and cell division, which was probably related to the excessive proliferation of cancer cells. The downregulated DEGs were associated with various metabolic processes and complement cascade, showing an obvious alteration in HCC metabolism.

**TABLE 1:** KEGG pathway enrichment analysis of upregulated DEGs.

Pathway	Name	Count	P Value	Genes
hsa04110	Cell cycle	7	1.32E-06	CDC20; CCNB1; CHEK1; CDK1; MCM4; TTK; MAD2L1
hsa04115	p53 signaling pathway	5	4.34E-05	CCNB1; RRM2; CHEK1; CDK1; GTSE1
hsa04114	Oocyte meiosis	5	3.11E-04	CDC20; CCNB1; CDK1; MAD2L1; AURKA
hsa04914	Progesterone-mediated oocyte maturation	3	0.028416	CCNB1; CDK1; MAD2L1

**TABLE 2:** REACTOME pathway enrichment analysis of DEGs.

Term	Description	Count	P Value	Genes
Upregulated				
R-HSA-69278	Cell Cycle, Mitotic	25	1.11E-16	TOP2A; GMNN; NCAPG; MCM10; HMMR; FOXM1; CENPA; AURKA; CDC20; CCNB1;…
R-HSA-1640170	Cell Cycle	28	1.11E-16	TOP2A; GMNN; HJURP; NCAPG; MCM10; HMMR; FOXM1; CENPA; AURKA; CDC20;…
R-HSA-453279	Mitotic G1 phase and G1/S transition	9	3.75E-12	TOP2A; CCNB1; RRM2; GMNN; CDK1; MCM4; MCM10; MYBL2; KIF23
R-HSA-69275	G2/M Transition	11	6.70E-10	TPX2; CCNB1; CDK1; IGF2BP3; KIF23; MYBL2; NEK2; HMMR; FOXM1; GTSE1; AURKA
R-HSA-453274	Mitotic G2-G2/M phases	11	7.49E-10	TPX2; CCNB1; CDK1; IGF2BP3; KIF23; MYBL2; NEK2; HMMR; FOXM1; GTSE1; AURKA
R-HSA-69620	Cell Cycle Checkpoints	14	1.89E-09	MCM10; KIF23; CENPA; NDC80; CDC20; CCNB1; CHEK1; CDK1; CENPM; BIRC5; MCM4;…
R-HSA-69206	G1/S Transition	7	3.29E-08	CCNB1; RRM2; GMNN; CDK1; MCM4; KIF23; MCM10
R-HSA-453276	Regulation of mitotic cell cycle	7	1.14E-07	CDC20; CCNB1; CDK1; KIF23; NEK2; AURKA; MAD2L1
R-HSA-174143	APC/C-mediated degradation of cell cycle proteins	7	1.14E-07	CDC20; CCNB1; CDK1; KIF23; NEK2; AURKA; MAD2L1
R-HSA-1538133	G0 and Early G1	3	1.53E-07	TOP2A; CDK1; MYBL2
Downregulated				
R-HSA-166658	Complement cascade	13	1.41E-09	FCN2; FCN3; CFP; C8A; C6; IGKC; C7; C9; CFHR4; IGLV1-44; CFHR5; IGLC1; MASP2
R-HSA-5661231	Metallothioneins bind metals	6	6.29E-08	MT1M; MT1F; MT1G; MT1H; MT1X; MT1E
R-HSA-211897	Cytochrome P450 - arranged by substrate type	12	1.02E-07	CYP39A1; CYP2C9; CYP2A7; CYP26A1; CYP2A6; CYP2C8; CYP2B6; CYP1A2; CYP4A11;…
R-HSA-211945	Phase I - Functionalization of compounds	15	2.25E-07	ADH1C; ADH1B; CYP4A11; CYP4A22; FMO3; CYP3A4; CYP39A1; CYP2C9; CYP2A7;…
R-HSA-5660526	Response to metal ions	6	3.06E-07	MT1M; MT1F; MT1G; MT1H; MT1X; MT1E
R-HSA-211859	Biological oxidations	20	3.30E-07	NNMT; ADH1C; ADH1B; CYP4A11; CYP4A22; ACSM5; GLYAT; FMO3; CYP3A4; CYP39A1...
R-HSA-9006931	Signaling by Nuclear Receptors	11	3.62E-07	CETP; CYP26A1; ADH1C; APOC2; APOC4; RDH16; RDH5; FOSB; FOS; PCK1; ESR1
R-HSA-211999	CYP2E1 reactions	5	4.00E-05	CYP2C9; CYP2A7; CYP2A6; CYP2C8; CYP2B6
R-HSA-2142753	Arachidonic acid metabolism	8	4.67E-05	CYP2C9; CYP2C8; CYP1A2; PON1; CYP4A11; CYP4A22; CYP1A1; PTGS2
R-HSA-1430728	Metabolism	54	0.001759957	CDA; VIPR1; CYP4A22; STAB2; ACSM5; DBH; GBA3; GYS2; ACADL; TDO2; HGFAC; SDS;…

### The representative gene sets in GSEA analysis

**FIGURE 3:**
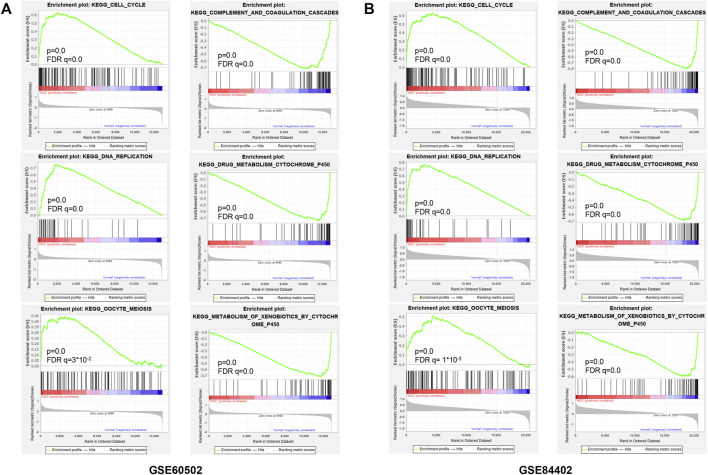
Enrichment plots from GSEA. Six representative functional gene sets enriched of HCC in GSE60502 **(A)** and GSE84402 **(B)**, including cell cycle, DNA replication, oocyte meiosis, complement and coagulation, drug metabolism cytochrome P450, and metabolism of xenobiotics by cytochrome P450.

For observing the overall functional enrichment of genes from liver cancer tissues and corresponding normal liver tissues respectively, the GSEA analysis was performed. As shown in Figures 3A,B, the representative gene sets enriched in the HCC group contained cell cycle, DNA replication, and oocyte meiosis. Similarly, these representative gene sets enriched in the normal group were complement and coagulation cascades, drug metabolism P450, and metabolism of xenobiotics by cytochrome P450 in both the GSE60502 dataset and GSE84402 dataset. Compared with normal liver tissues, upregulated genes were primarily associated with cell cycle and cell division in these two datasets, while downregulated genes were mainly related to substance metabolism and the complement cascade.

### Protein-protein interaction network analysis of differentially expressed genes

**FIGURE 4:**
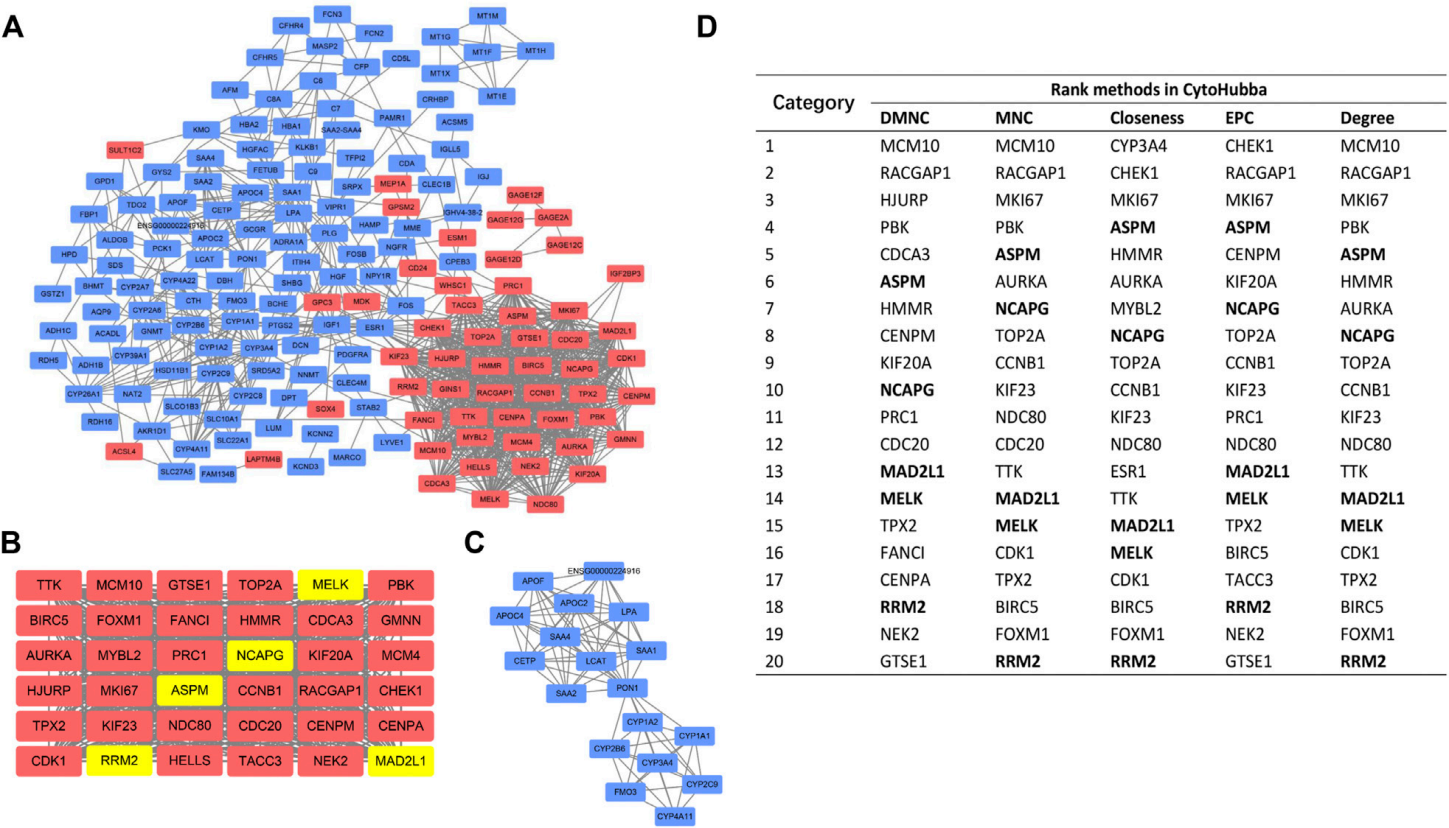
PPI network construction and analyses. **(A)** The whole PPI network contained 170 DEGs (54 upregulated DEGs labeled in red and 116 downregulated DEGs labeled in blue). **(B)** PPI network of module 1. **(C)** PPI network of module 2. **(D)** Hub genes ranked by five CytoHubba methods. Bold gene symbols were the overlapped genes in the top 20 by five ranking methods.

We constructed the PPI network by Cytoscape software on the basis of the STRING database, which consisted of 170 nodes and 1056 edges (Figure 4A). Next, two important modules were obtained from the network using MCODE in Cytoscape. Module 1 contained 36 nodes and 607 edges with a score of 34.686 (Figure 4B); Module 2 contained 18 nodes and 90 edges with a score of 9.412 (Figure 4C). Surprisingly, genes in module 1 were all upregulated, while these in module 2 were downregulated. KEGG pathway enrichment analysis of DEGs from two modules showed that the DEGs in module 1 were mainly enriched in cell cycle, p53 signaling pathway, oocyte meiosis, and progesterone-mediated oocyte maturation (Table 3), and DEGs in module 2 were mainly enriched in retinol metabolism, drug metabolism-cytochrome P450, metabolism of xenobiotics by cytochrome P450, chemical carcinogenesis, linoleic acid metabolism (Table 4). The results indicated that module 1 DEGs were significant in the entire PPI network. Similarly, DEGs from module 2 occupied an important position in all downregulated DEGs. Then, RRM2, MAD2L1, MELK, NCAPG, and ASPM were selected as hub genes, which scored in the top 20 by all five methods in CytoHubba (Figure 4D). Remarkably, these upregulated genes were all in module 1, implying that these five genes may play a pivotal role in HCC development.

**TABLE 3:** KEGG pathway enrichment analysis of Module 1 genes function.

Term	Description	Count	P Value	Genes
hsa04110	Cell cycleact	7	8.11E-08	CDC20; CCNB1; CHEK1; CDK1; MCM4; TTK; MAD2L1
hsa04115	p53 signaling pathway	5	7.65E-06	CCNB1; RRM2; CHEK1; CDK1; GTSE1
hsa04114	Oocyte meiosis	5	5.67E-05	CDC20; CCNB1; CDK1; MAD2L1; AURKA
hsa04914	Progesterone-mediated oocyte maturation	3	0.013036882	CCNB1; CDK1; MAD2L1

**TABLE 4:** KEGG pathway enrichment analysis of Module 2 genes function.

Term	Description	Count	P Value	Genes
hsa00830	Retinol metabolism	5	1.01E-07	CYP2C9; CYP2B6; CYP1A2; CYP1A1; CYP3A4
hsa00982	Drug metabolism - cytochrome P450	5	1.29E-07	CYP2C9; CYP2B6; CYP1A2; FMO3; CYP3A
hsa00980	Metabolism of xenobiotics by cytochrome P450	5	1.82E-07	CYP2C9; CYP2B6; CYP1A2; CYP1A1; CYP3A4
hsa05204	Chemical carcinogenesis	4	2.95E-05	CYP2C9; CYP1A2; CYP1A1; CYP3A4
hsa00591	Linoleic acid metabolism	3	2.55E-04	CYP2C9; CYP1A2; CYP3A4
hsa00140	Steroid hormone biosynthesis	3	0.001025554	CYP1A2; CYP1A1; CYP3A4
hsa01100	Metabolic pathways	5	0.010872421	CYP2C9; CYP2B6; CYP1A2; CYP1A1; CYP3A4
hsa00380	Tryptophan metabolism	2	0.03439785	CYP1A2; CYP1A1

### Validation of the expression of 5 hub genes in hepatocellular carcinoma

**FIGURE 5:**
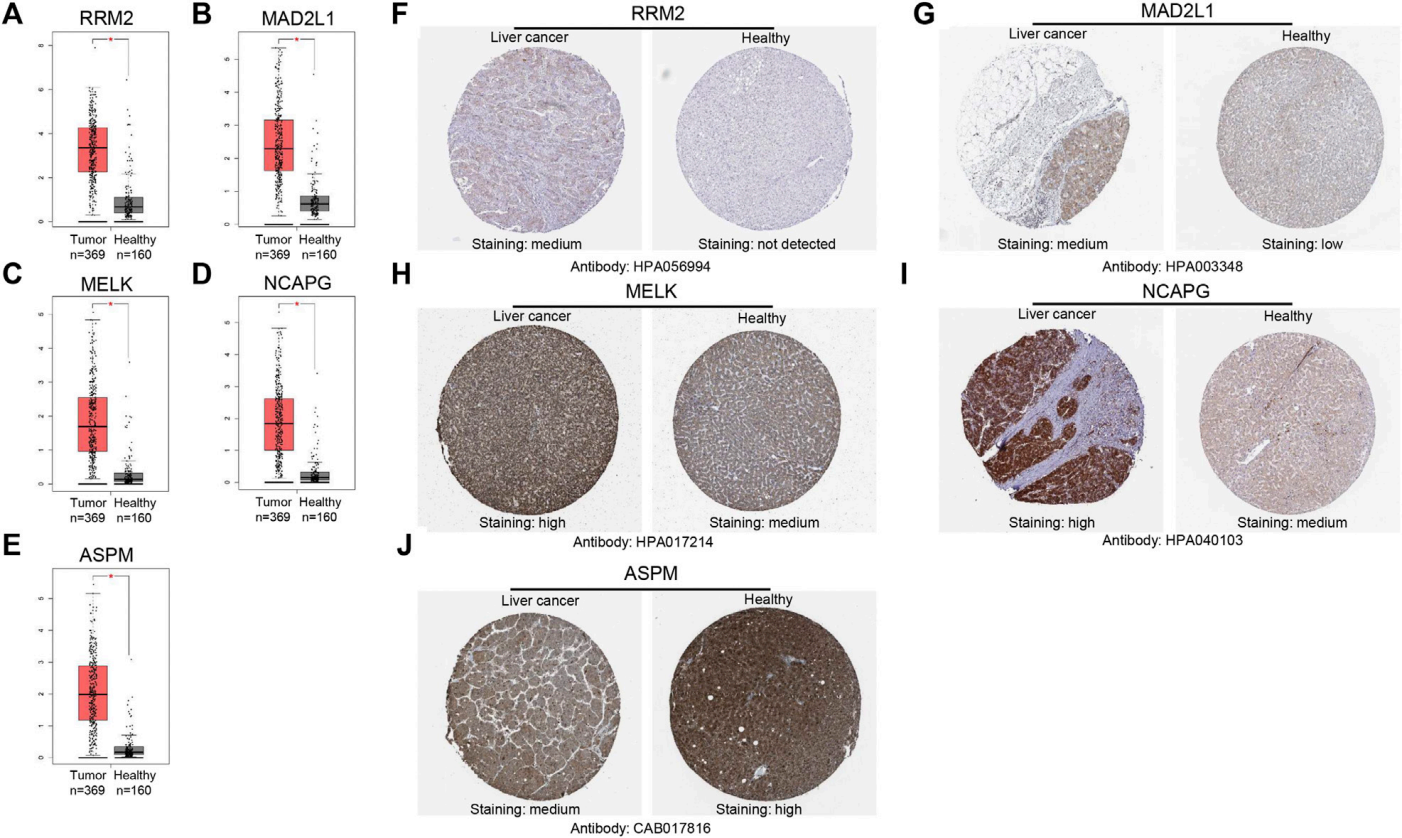
Validation of the mRNA expression levels of **(A)** RRM2, **(B)** MAD2L1, **(C)** MELK, **(D)** NCAPG, **(E)** ASPM in HCC tissues compared with normal liver tissues using GEPIA. **(F–J)** Immunohistochemistry (IHC) validation about these genes in HCC tissues and liver tissues.

The GEPIA database was used to validate the mRNA expression levels of the above five hub genes in HCC, and the results revealed that all hub genes were highly expressed in HCC tissues compared with normal liver tissues (Figures 5A–E), which were consistent with the obtained microarray data. Then, we explored the protein expression levels of these genes by using the HPA database. As shown in (Figures 5F–J), the protein expression levels of RRM2, MAD2L1, MELK, and NCAPG in HCC tissues were higher than those in normal liver tissues. The high protein expression level of ASPM was observed in normal liver tissues, and that in liver cancer tissues was medium. Overall, the mRNA and protein expression levels of these genes were overexpressed in cancer tissues compared with corresponding normal liver tissues.

### Immune infiltration analysis and survival analysis of hub genes

**FIGURE 6:**
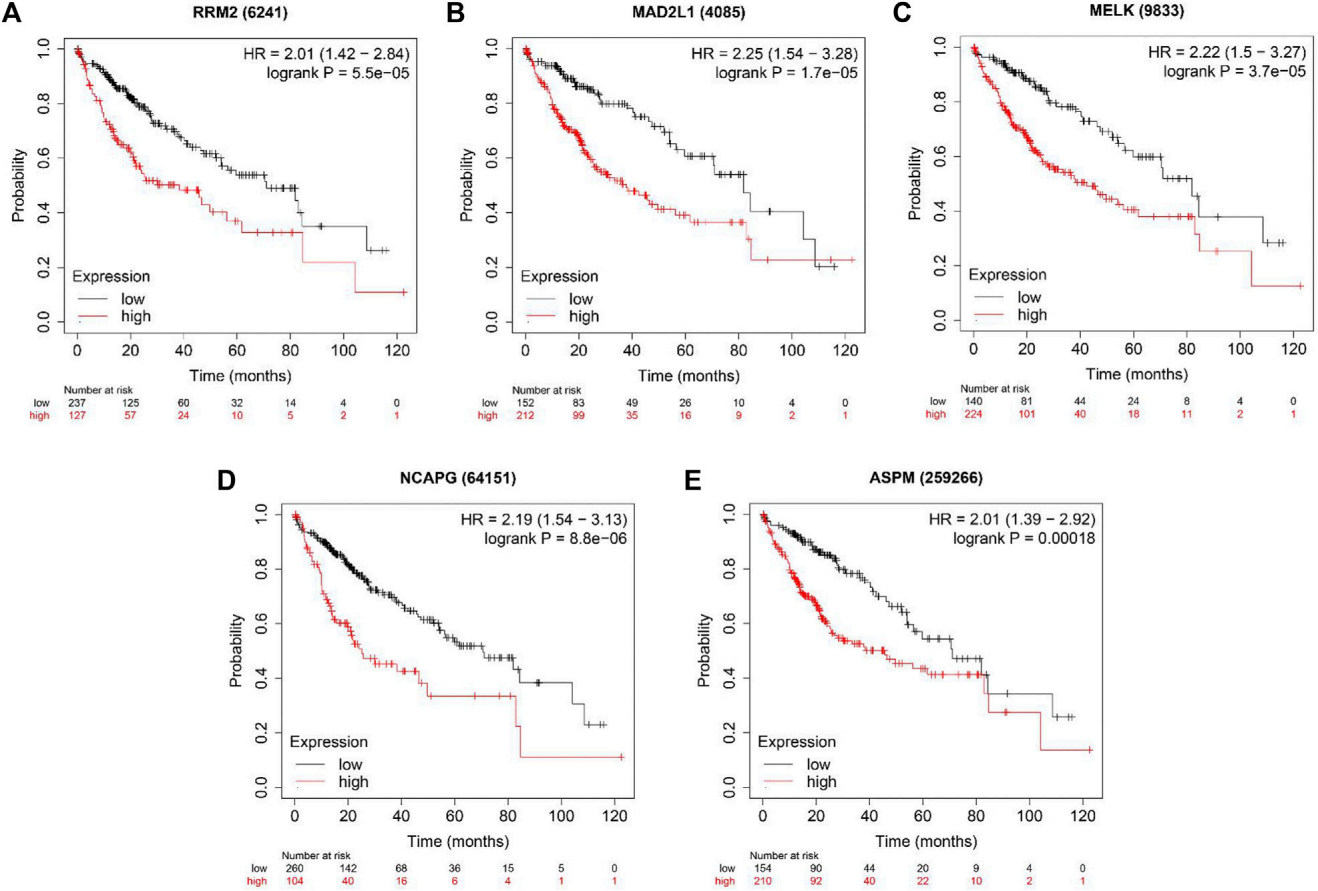
The OS analysis of 5 hub genes in the HCC tissues by Kaplan–Meier plotter. **(A)** RRM2; **(B)** MAD2L1; **(C)** MELK; **(D)** NCAPG and **(E)** ASPM.

We used the TIMER database to evaluate whether expression levels of these genes could affect immune cell infiltration. As shown in Supplementary Figures S2A–E, the expression levels of these five genes were positively associated with the infiltration of B cells, CD8^+^ T cells, CD4^+^ T cells, macrophage cells, neutrophil cells and dendritic cells. However, all five hub genes did not correlate with purity. Furthermore, overall survival (OS) analyses of the five genes were conducted using Kaplan-Meier plotter. As shown in Figure 6, high expression of RRM2, MADAL1, MELK, NCAPG and ASPM were associated with poor OS for liver cancer patients.

### RRM2 inhibitor osalmid inhibited proliferation and migration, promoted cell apoptosis, triggered cell cycle arrest, and induced DNA damage of hepatocellular carcinoma cells.

In many malignant tumors, the expression level of RRM2 is increased to promote rapid proliferation of cancer cells, and RRM2 inhibition has gradually became a practical cancer treatment strategy (Shao et al., 2013; Aye et al., 2015). As a novel RRM2 inhibitor, osalmid repressed RRM2 activity by competitive binding to RRM2 hydrogen bond active site. Its unique molecular structure could enhance the binding affinity to RRM2, so as to forming more specific RRM2 inhibitor (Liu et al., 2016). It was 10 times more active than hydroxyurea in inhibiting ribonucleotide reductase activity by targeting RRM2, and inhibited HBV genomic DNA and the viral covalently closedcircular DNA (cccDNA) synthesis. Furthermore, it has been proven that osalmid has minimal cytotoxicity to be a superior RRM2 inhibitor (Liu et al., 2016).

**FIGURE 7:**
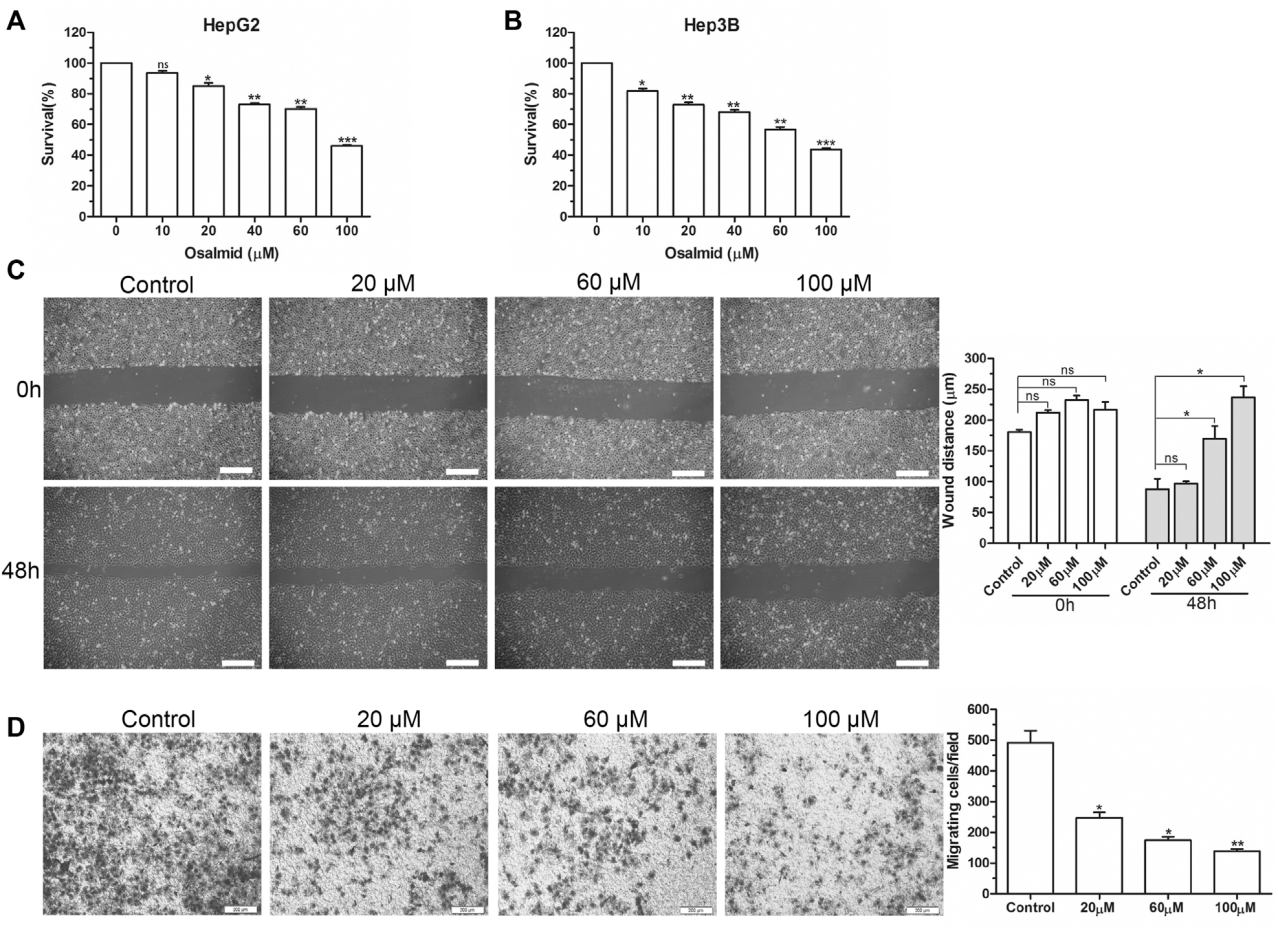
The effect of RRM2 inhibitor osalmid on proliferation and migration of HCC cells. **(A,B)** CCK-8 assay analysis of the cell viabilities in HepG2 and Hep3B cells treated with different concentrations of osalmid for 48 h. Scratch test **(C)** and Transwell experiment **(D)** showed that Hep3B cell migration ability was inhibited with different doses of osalmid after 48 h. Scale bar = 200 μm. Error bars, mean ± SD. ns, non significant. *, *p* < 0.05; **, *p* < 0.01; ***, *p* < 0.001.

**FIGURE 8:**
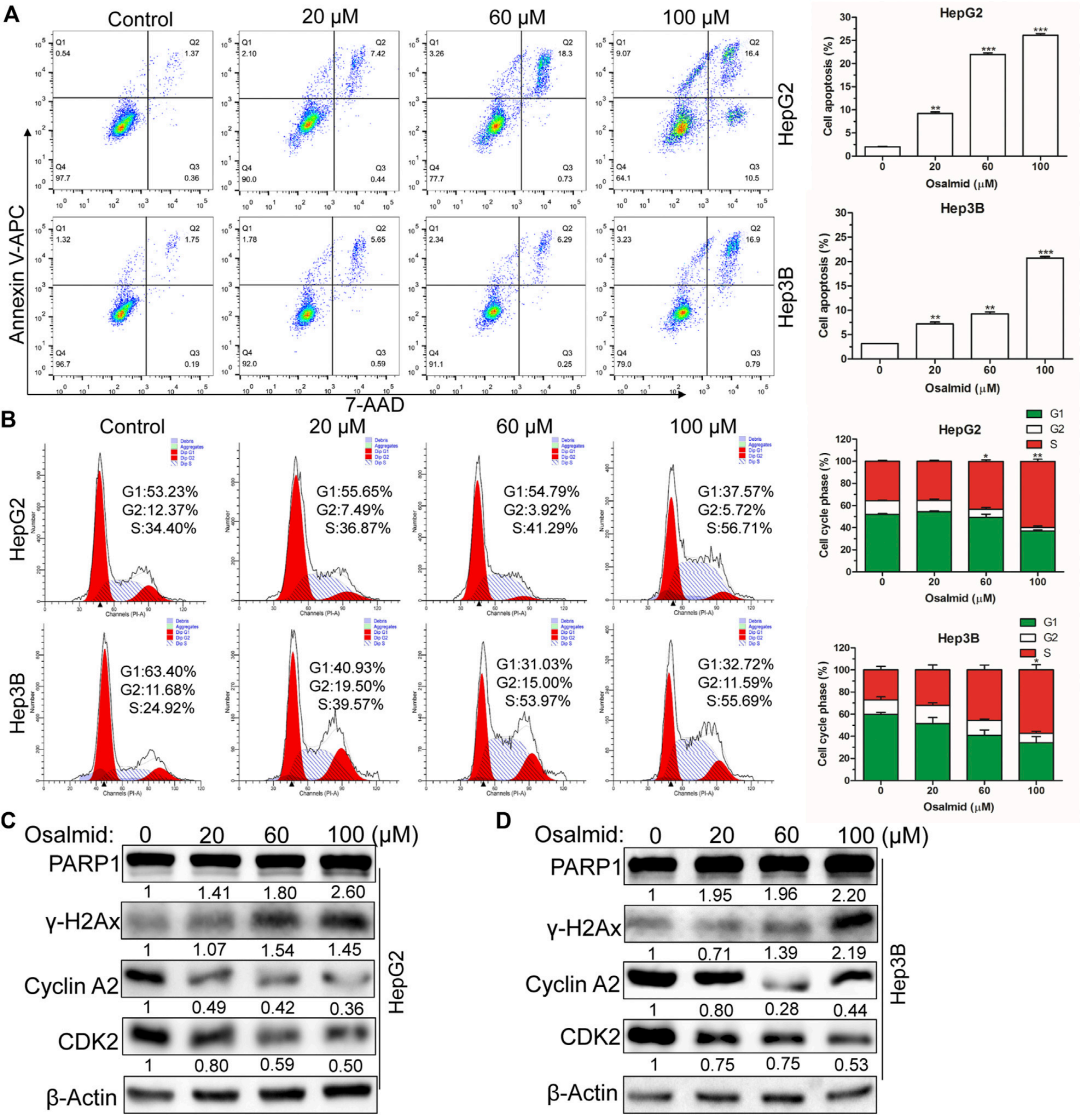
Osalmid promoted cell apoptosis, triggered cell cycle S phase arrest, and induced DNA damage of HCC cells. After treatment with osalmid for 48 h, the cell apoptosis rate **(A)** and cell cycle distribution **(B)** were analyzed by flow cytometry in HCC cells. **(C,D)** Western blot analyses of the protein expression of PARP1, cyclinA2, CDK2, and γH2AX in HepG2 and Hep3B cells, respectively. β-Actin was used as the internal reference. The experiments were repeated three times. The bands were quantified using ImageJ software. Error bars, mean ± SD. *, *p* < 0.05; **, *p* < 0.01; ***, *p* < 0.001.

Firstly, a CCK-8 cell viability assay was used to evaluate the cytotoxic effect of osalmid on HCC cells, and the results suggested that osalmid dose-dependently inhibited HCC cell viabilities (Figures 7A,B). We conducted a wound-healing and Transwell migration assay to investigate whether osalmid could affect the migration of HCC cells. The wound assay revealed that the migration ability of Hep3B cells was significantly suppressed following osalmid treatment for 48 h (Figure 7C). This was further verified by the Transwell migration experiment in which the amount of migrated Hep3B cells markedly decreased in a concentration-dependent manner (Figure 7D). Next, we further explored whether osalmid had an impact on HCC cell death and cell cycle distribution. As shown in Figure 8A, osalmid increased cell apoptosis in a dose-dependent manner. And poly (ADP-ribose) polymerase 1 (PARP1) protein was activated when HepG2 and Hep3B cells were treated with different dose of osalmid (Figures 8C,D). The analysis of the cell cycle showed that the percentage of S phase dose-dependently increased after osalmid treatment (Figure 8B). Further, western blot demonstrated that the expression of S-phase cyclinA2 and CDK2 were inhibited, which was consistent with flow cytometry results (Figures 8C,D). As a small subunit of ribonucleotide reductase, RRM2 is required for DNA synthesis, then we detected expression of γH2AX which is the hallmark of DNA damage. As expected, the expression of γH2AX was activated by osalmid in a dose-dependent manner (Figures 8C,D). To summarize, RRM2 may be a novel therapeutic target for HCC patients.

## Discussion

Hepatocellular carcinoma is a highly aggressive malignancy with rapid development, low rate of early diagnosis, and dismal prognosis, whose risk factors include hepatitis B and C infection, liver cirrhosis, aflatoxin exposure, etc (Lin et al., 2017; Lou et al., 2018; Jiang et al., 2020). Though many related studies on HCC have been conducted, early diagnosis, therapeutic effects, and prognosis have not been well resolved. Further understanding of the molecular mechanisms resulting in occurrence and development is critical for diagnosis and treatment. With the development of high–throughput sequencing technology, the usage of bioinformatics tools to recognize biological markers has been prevalent.

In this study, we initially analyzed the expression of genes in two microarray datasets and identified 215 common DEGs (71 upregulated and 144 downregulated). Next, a series of bioinformatics analyses were performed to explore these DEGs deeply. Enrichment analyses revealed that the upregulated DEGs were mainly associated with DNA replication, cell cycle, and cell division, which could account for the excessive proliferation of cancer cells reasonably. The functions of downregulated DEGs were closely related to various metabolic processes and the complement cascade, suggesting that the metabolic process of HCC cells had changed. This was possible because tumor cells may not have normal metabolic functions. A PPI network of DEGs was constructed subsequently to determine potential “key” genes, we identified five key genes for the occurrence and development in HCC, including RRM2, MAD2L1, MELK, NCAPG, and ASPM. It has been reported that immune cell infiltration is associated with proliferation and progression of cancer cells (Li et al., 2020a; Chen and Zhang, 2020). The TIMER database was used to explore the correlation between the five genes and immune cell infiltration. There was a merely weak partial correlation with diverse immune cells, including B cells, CD8^+^ T cells, CD4^+^ T cells, macrophage cells, neutrophil cells, and dendritic cells, and all five hub genes were not associated with purity. Hence, the relationship between these genes and immune cells needs to be further explored. Finally, we found that the high expression levels of these five genes were related to poor overall survival in HCC patients based on the Kaplan-Meier plotter.

As a new oncogene, MAD2L1 had been studied in detail in oncogenic contexts (Li et al., 2017c; Li et al., 2020b; Ding et al., 2020). Lin et al. (2017) reported that MAD2L1 was significantly higher expressed in combined hepatocellular and intrahepatic cholangiocarcinoma tissues. Li et al. (2017c) found that MAD2L1 promoted HCC cell viabilities, while suppressing MAD2L1 expression by miR-200c-5p could inhibit the proliferation, migration, invasion and induce apoptosis and cell cycle arrest of HCC cells. It was reported that the expression level of MELK in HCC was extremely higher than that in in other types of cancer (Hiwatashi et al., 2016). Xia et al. (2016) reported that cell growth, invasion, stemness and tumorigenicity of HCC cells could be inhibited by silencing MELK, suggesting that MELK was an oncogenic kinase involved in the pathogenesis and recurrence of HCC. Furthermore, drug inhibition on MELK could also suppress tumor growth, implying this kinase will be a therapy target (Chlenski et al., 2019). NCAPG was involved in the pathogenesis of a variety of tumors, including prostate cancer, renal cell carcinoma, multiple myeloma, melanoma and HCC, leading to inferior survival of patients (Gong et al., 2019; Xiao et al., 2020). In HCC, the overexpression of NCAPG could activate the PI3K/AKT/FOXO4 pathway, promoting cell proliferation and reducing cell apoptosis (Gong et al., 2019). Meanwhile, NCAPG could be a prognostic biomarker for vascular invasion, and high levels of NCAPG expression was linked to poor survival outcomes (Guo and Zhu, 2021). Lin et al. demonstrated that the overexpression of ASPM in HCC was a reliable marker for early tumor recurrence, contributing to distant metastasis and poor prognosis (Lin et al., 2008). Our results showed that these four genes were all upregulated DEGs and resulted in poor prognosis of patients, which were consistent with the above researches. Nevertheless, no further research has been done on these genes in our study. Subsequent studies can explore the role of these genes in HCC.

RRM2, a rate-limiting enzyme functioning in the formation of ribonucleotides into deoxyribonucleotides, is very important for DNA replication (Koppenhafer et al., 2020), which is overexpressed in various tumor progression, leading to poor prognoses (Li et al., 2018; Mazzu et al., 2019; Jin et al., 2020). Lu et al. (2012) established that RRM2 expression was relevant to depth of invasion, poor differentiation, and tumor metastasis in colorectal cancer. Studies have shown that RRM2 could regulate anti-tumor immune response, and RRM2 knockout could improve the anti-tumor efficiency of PD-1 blocker in renal cancer, implying that RRM2 may be a promising therapeutic target for renal cell carcinoma (Xiong et al., 2021). It has been widely recognized that DNA replication can precisely replicate the genetic information of cells and transmit it to offspring cells. RRM2, as a hub gene in HCC, was a key enzyme of DNA replication to regulate cell proliferation and cell cycle. But the detailed molecular role in HCC remains unclear. So biological experiments were performed to further explore the mechanism of RRM2 in HCC. We used a specific RRM2 inhibitor osalmid to inhibit RRM2 expression and then evaluated its effect on HCC progression. The experimental results indicated that osalmid could inhibit proliferation and migration, promote cell apoptosis, block cell cycle and induce DNA damage to HCC cells. Nevertheless, our research still had some limitations: 1) the sample size was not large enough; 2) sample specificity was not considered; 3) whether RRM2 was related to the sensitivity of HCC to chemotherapy drugs has been unclear; 4) more detailed molecular mechanisms needed to be elucidated.

## Conclusion

Using a variety of bioinformatics, we identified some critical signaling pathways and five hub genes (RRM2, MAD2L1, MELK, NCAPG, and ASPM) related to the pathogenesis and progression of HCC. With the RRM2 inhibitor osalmid, the biological effects of silencing RRM2 expression on HCC were determined through biological experiments, thus proving that targeting RRM2 may become a new strategy for the treatment of HCC patients.

## Data Availability

The original contributions presented in the study are included in the article/Supplementary Material, further inquiries can be directed to the corresponding author.
