# Supplementary figures and images for: Identifying potential prognosis markers in hepatocellular carcinoma via integrated bioinformatics analysis and biological experiments
Source: Front Genet. 2022 Jul 19;13:942454. doi: 10.3389/fgene.2022.942454 (PMC9343963; doi:10.3389/fgene.2022.942454)

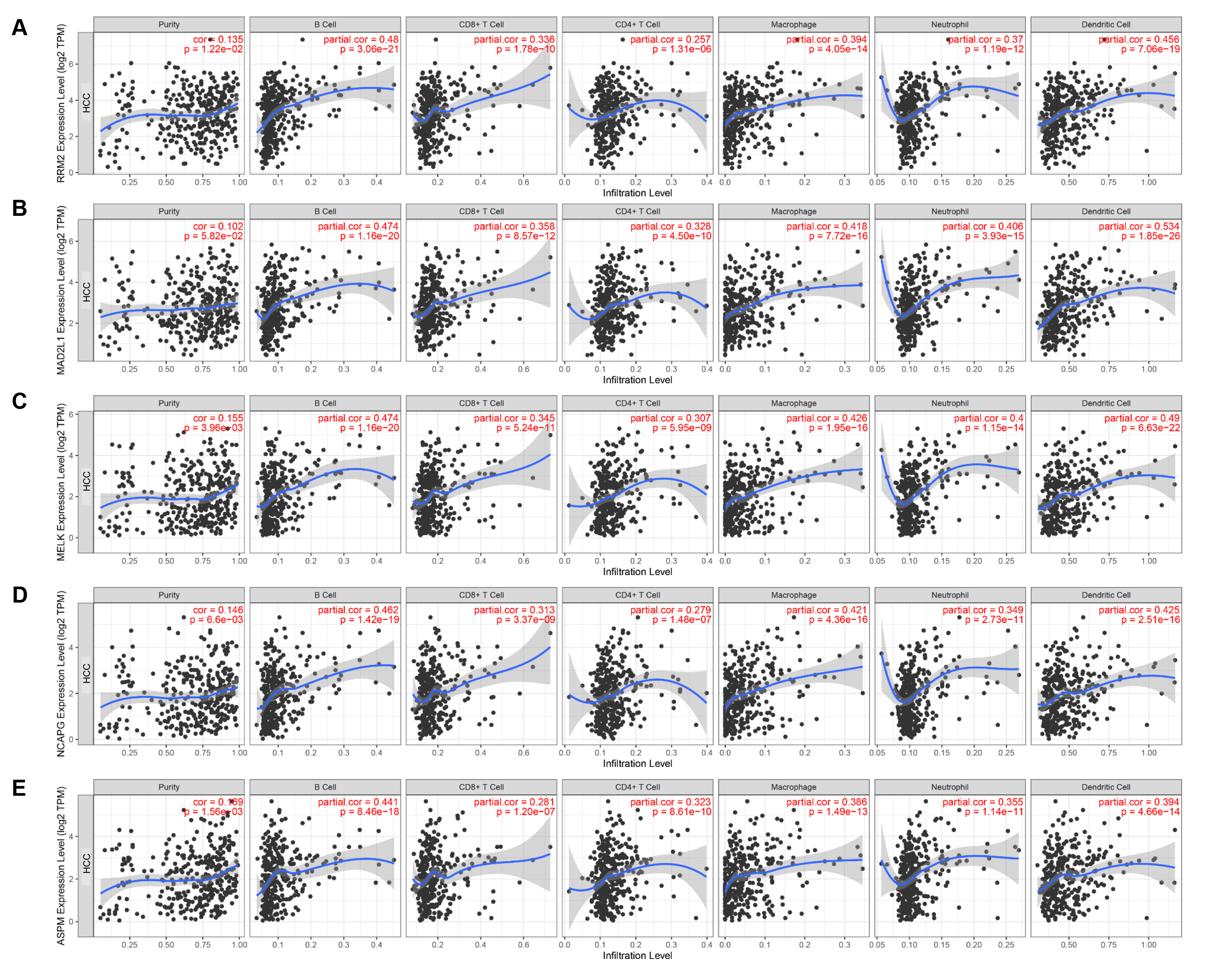

Supplement: Supplementary file 1 [file Image2.TIF]

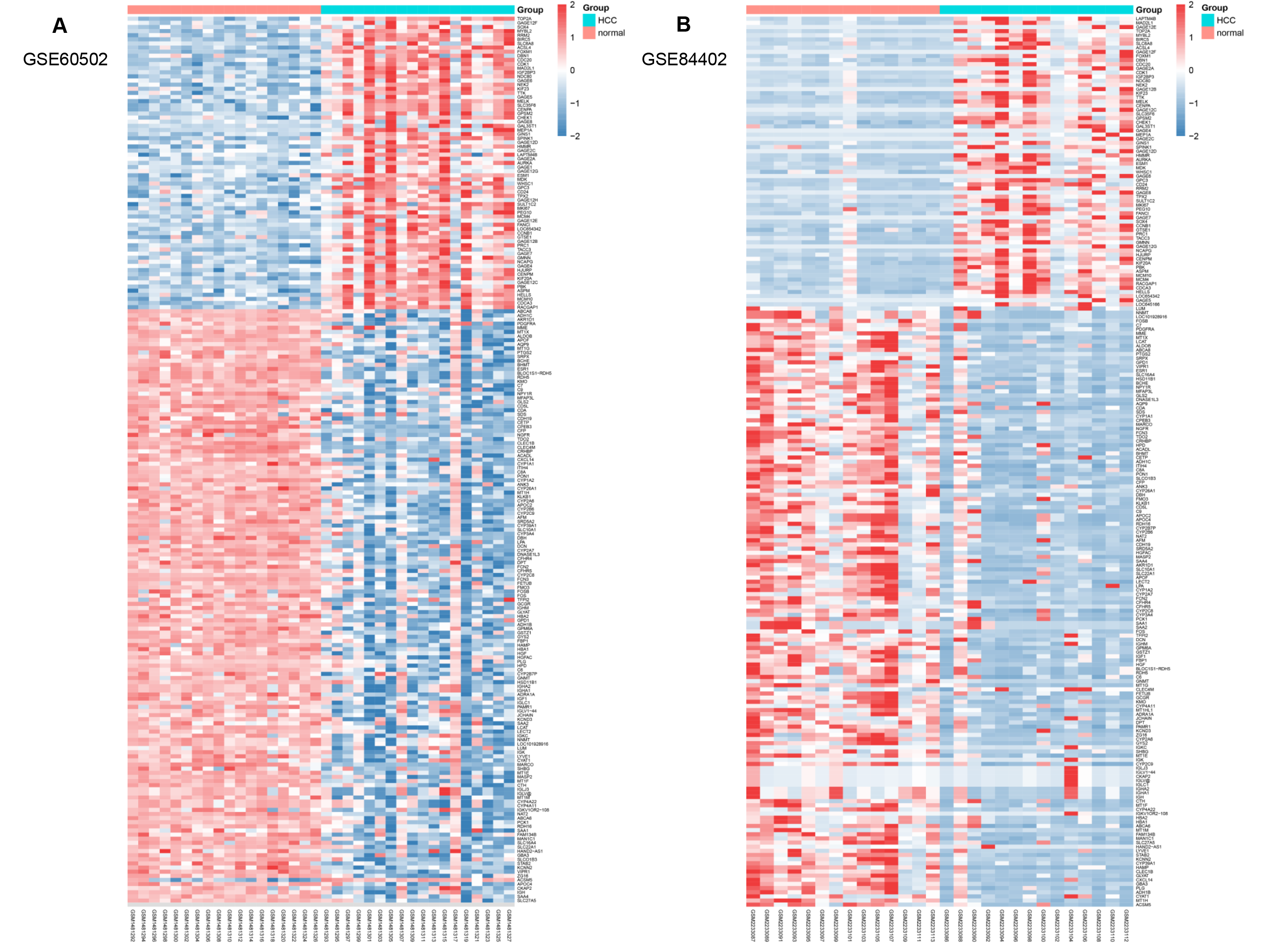

Supplement: Supplementary file 2 [file Image1.TIF]
